# Cardiovascular Complications of Diabetes: From Microvascular to Macrovascular Pathways

**DOI:** 10.7759/cureus.45835

**Published:** 2023-09-24

**Authors:** Mehreen Zakir, Neha Ahuja, Marvi Alais Surksha, Reya Sachdev, Yash Kalariya, Muhammad Nasir, Maham Kashif, Fnu Shahzeen, Arslan Tayyab, Muhammad Saood moazzam Khan, Mohsin Junejo, FNU Manoj Kumar, Giustino Varrassi, Satesh Kumar, Mahima Khatri, Tamam Mohamad

**Affiliations:** 1 Medicine, Sir Syed College of Medical Sciences, Karachi, PAK; 2 General Medicine, Chandka Medical College, Larkana, PAK; 3 Medicine, Ghulam Muhammad Mahar Medical College, Sukkur, PAK; 4 Internal Medicine, Civil Hospital Rajkot, Rajkot, IND; 5 Medicine, Rural Health Center (RHC) Dhonkal, Dhonkal, PAK; 6 Medicine, Khawaja Muhammad Safdar Medical College, Sialkot, PAK; 7 Internal Medicine, Jinnah Sindh Medical University, Karachi, PAK; 8 Internal Medicine, Quaid e Azam Medical College, Bahawalpur, PAK; 9 Medicine, Chandka Medical College, Larkana, PAK; 10 Medicine, Jinnah Sindh Medical University, Karachi, PAK; 11 Pain Medicine, Paolo Procacci Foundation, Rome, ITA; 12 Medicine and Surgery, Shaheed Mohtarma Benazir Bhutto Medical College, Karachi, PAK; 13 Medicine and Surgery, Dow University of Health Sciences, Karachi, PAK; 14 Cardiovascular Medicine, Wayne State University, Detroit, USA

**Keywords:** macrovascular, microvascular, cvd, cardiovascular, diabetes

## Abstract

Diabetes mellitus, with a growing risk of developing complications, has a significant negative impact on cardiovascular health, including microvascular and macrovascular issues. This thorough narrative study methodically examines the complex connection between cardiovascular problems and diabetes. We start by thoroughly introducing diabetes mellitus, classifying its various forms, and discussing its growing global impact. Then, we examine retinopathy, nephropathy, and neuropathy in detail, illuminating their biology, clinical presentations, and treatment options. Moving on to macrovascular consequences, we investigate the complex relationships between diabetes and coronary artery disease, stroke, and peripheral arterial disease, emphasizing risk factors, diagnostic standards, and treatment plans designed for people with diabetes. The review analyzes the pathophysiological pathways that link diabetes to cardiovascular problems, including endothelial dysfunction, chronic inflammation, immune system dysregulation, and oxidative stress brought on by hyperglycemia. Additionally, we review the critical function of risk monitoring, assessment, and predictive tools in early detection. While highlighting current research paths and the need for tailored medical approaches to address this complex health issue, the story also includes prevention and management strategies, ranging from lifestyle changes to developing medications. This narrative review concludes by providing a thorough summary of current information, highlighting research gaps, and advocating for interdisciplinary efforts to reduce the cardiovascular effects of diabetes.

## Introduction and background

Diabetes mellitus, commonly known as diabetes, has become a significant global health concern in the 21st century. A complex impact accompanies the increasing prevalence of diabetes on both health systems and individual well-being. The diabetes epidemic is characterized by complex complications, particularly those that affect the cardiovascular system. These complications pose a significant burden beyond geographical boundaries [1]. This narrative review aims to examine the complexities surrounding cardiovascular complications in diabetes. It will navigate the intricate landscape of these complications, encompassing both microvascular and macrovascular pathways. Diabetes has emerged as a significant global health concern, with its prevalence reaching pandemic proportions. Diabetes is a chronic metabolic disorder with elevated blood glucose levels [2]. It is recognized as one of the most prevalent non-communicable diseases globally. The exponential growth observed in recent decades has elevated it to a global health priority. Our most recent knowledge update in September 2021 estimated that around 10% of the global population, equivalent to approximately 463 million individuals, were living with diabetes. The projected number of individuals affected by diabetes is expected to reach 700 million by 2045 if current trends continue. This alarming statistic highlights the significant global health challenge of diabetes, which has far-reaching implications for healthcare systems, economies, and overall quality of life [3].

The impact of diabetes extends beyond its immediate metabolic abnormalities. Diabetes encompasses more than just increased blood sugar levels. It is a systemic condition that affects various organ systems, leading to complications that significantly impact morbidity and mortality rates [4]. Cardiovascular diseases are of utmost significance among the various complications, emphasizing the crucial requirement for a comprehensive comprehension of these interconnected ailments' mechanisms, risks, and management. The cardiovascular complications associated with diabetes, commonly called "diabetic cardiomyopathy," encompass a range of disorders that pose significant challenges within modern medicine. The relationship between diabetes and cardiovascular complications is not limited to a single pathophysiological pathway. However, it involves a complex interaction of factors that affect both microvascular and macrovascular structures [5]. The microvascular complications associated with diabetes, including retinopathy, nephropathy, and neuropathy, indicate the progressive and severe impact of the disease. Diabetic retinopathy is a prevalent cause of adult blindness, characterized by the gradual deterioration of blood vessels in the retina [6]. Diabetic nephropathy, however, gradually impairs kidney function, ultimately leading to end-stage renal disease in many patients. In the meantime, diabetic neuropathy significantly impacts the peripheral nerves, resulting in pain, sensory impairments, and potential limb amputations. Due to their asymptomatic progression, the microvascular complications significantly impact the quality of life and healthcare resources [6].

In addition to its impact on the microvascular system, diabetes has detrimental effects on the macrovascular system, leading to the development of atherosclerosis, coronary artery disease (CAD), stroke, and peripheral arterial disease (PAD). Individuals diagnosed with diabetes are at a significantly increased risk of developing coronary artery disease (CAD), as the walls of their arteries are more prone to forming atherosclerotic plaques faster [7]. This leads to an increased prevalence of myocardial infarctions and unstable angina. Diabetes also increases the risk of stroke, including ischemic and hemorrhagic types, which further impact the cerebrovascular system. Furthermore, individuals with diabetes are likely to experience peripheral arterial disease, a condition characterized by compromised blood flow to the extremities. This condition is not only more prevalent but also tends to advance at a faster rate in diabetic patients [7]. These macrovascular complications play a significant role in cardiovascular morbidity and mortality among individuals with diabetes [5-7]. It is crucial to comprehend the complexities of the interconnection between microvascular and macrovascular pathways in cardiovascular complications associated with diabetes. Acknowledging that these complications are not isolated incidents but components of a larger continuum enable a more comprehensive approach to prevention and management [8]. This narrative review explores these pathways' molecular and physiological complexities, providing insight into their pathogenesis, clinical manifestations, diagnostic criteria, and therapeutic approaches. The ongoing global prevalence of diabetes necessitates urgent attention to the multifaceted challenge posed by its associated cardiovascular complications. Understanding microvascular and macrovascular pathways is crucial to effectively addressing the escalating health crisis [9]. This narrative review aims to shed light on the intricate nature of cardiovascular complications associated with diabetes. We intend to create a comprehensive resource valuable to healthcare providers, researchers, and policymakers actively addressing the diabetes pandemic [6-10].

## Review

Diabetes mellitus: understanding the complexities of metabolism

Diabetes mellitus is a multifaceted and widespread metabolic disorder that impairs the body's ability to regulate blood glucose levels adequately [1]. Diabetes is fundamentally defined by chronic hyperglycemia, which refers to elevated levels of glucose in the bloodstream that surpass normal physiological thresholds. Metabolic dysregulation occurs due to insufficient insulin production, a hormone essential for the uptake and utilization of glucose, and the impaired responsiveness of body tissues to insulin. There are two main classifications of diabetes mellitus, namely Type 1 and Type 2 [2]. Each classification has unique pathophysiological foundations, risk factors, and clinical manifestations.

Type 1 diabetes: An autoimmune onslaught type 1 diabetes is commonly known as insulin-dependent diabetes or juvenile-onset diabetes. This condition is characterized by the autoimmune destruction of pancreatic beta cells, the specialized cells responsible for insulin production [3]. The immune-mediated attack causes a complete lack of insulin, resulting in a high dependence on external insulin for regulating blood sugar levels. Type 1 diabetes presents itself early in life, frequently during childhood or adolescence. However, it has the potential to develop at any point in time. The precise cause of this autoimmune response is currently unknown. However, genetic predisposition and environmental factors are widely considered significant contributing factors [4].

Type 2 Diabetes and the Challenge of Insulin Resistance

Type 2 diabetes, on the other hand, is distinguished by a distinct array of metabolic disruptions. This condition is commonly known as non-insulin-dependent diabetes or adult-onset diabetes. However, its incidence among younger individuals is increasing significantly. Insulin resistance is a defining characteristic of type 2 diabetes, whereby the cells in the body do not adequately respond to insulin signals, resulting in impaired glucose uptake [5]. Furthermore, the pancreas can experience a gradual decline in its capacity to produce an adequate amount of insulin, leading to a state of relative insulin deficiency. The onset of type 2 diabetes is ]intricately connected to a multifaceted interaction of genetic, environmental, and lifestyle elements, encompassing obesity, sedentary habits, and dietary preferences [6].

Escalating Prevalence and Incidence of the Global Diabetes Epidemic

The prevalence and incidence rates of diabetes have increased significantly, transforming it from a relatively rare condition to a global pandemic of great concern. The continuous increase in diabetes cases has established it as a significant public health issue in nearly every region. Based on the latest estimates as of September 2021, the global population of individuals with diabetes is approximately 463 million. It is important to note that this number is steadily increasing. Based on current trends, it is projected that the prevalence of diabetes cases could surpass 700 million by the year 2045. The statistics above emphasize the importance of comprehending the mechanisms that contribute to the diabetes epidemic and the extensive impact it has on healthcare systems and economies [7]. Although diabetes primarily impacts adults, there is a notable shift in the demographic landscape, as there is a worrisome trend of rising diabetes rates among younger populations. In the past, type 1 diabetes was commonly recognized as a condition predominantly diagnosed in the pediatric population, leading to its alternative designation as "juvenile-onset diabetes." Nonetheless, the classification of type 2 diabetes in children and adolescents is challenged by its increasing occurrence, often linked to obesity and sedentary behaviors [8]. The demarcation between these two categories is progressively becoming less distinct, indicative of the evolving epidemiology of diabetes.

The core of both type 1 and type 2 diabetes involves fundamental pathophysiological mechanisms that disturb glucose homeostasis. The central event in type 1 diabetes is the autoimmune destruction of pancreatic beta cells. The persistent assault initiated by the body's immune system is thought to arise from genetic predisposition and environmental stimuli, such as viral infections. When a significant number of beta cells are destroyed, there is a substantial reduction in insulin production, leading to hyperglycemia [9]. A complex interplay of various factors characterizes type 2 diabetes. Insulin resistance, which primarily manifests in muscle, liver, and adipose tissue, plays a central role in its pathogenesis. The resistance observed results from an intricate network of molecular events, encompassing abnormal signaling pathways, inflammatory mechanisms, and changes in lipid metabolism [10]. In response to insulin resistance, the pancreas initially increases insulin production, resulting in the condition known as hyperinsulinemia. Over time, there may be a decline in beta cell function, leading to a relative insulin deficiency. Dysfunctional adipose tissue also plays a role in the pathophysiology of diabetes by releasing pro-inflammatory cytokines and adipokines, which worsen insulin resistance and hinder glucose metabolism [8-10].

Dysfunction of Beta Cells: A Shared Factor

The primary pathology observed in Type 1 diabetes is the progressive destruction of beta cells. Conversely, in Type 2 diabetes, beta cell dysfunction is a significant factor that contributes to the development of the disease, in addition to insulin resistance [11]. The precise mechanisms underlying beta cell dysfunction are complex and involve various factors, such as genetic influences, glucotoxicity (adverse effects of elevated glucose levels on beta cells), lipotoxicity (adverse effects of excessive lipids on beta cells), and the accumulation of toxic protein aggregates. The dysfunction of beta cells worsens insulin deficiency, thereby further contributing to hyperglycemia in individuals with type 2 diabetes [12]. Diabetes mellitus is a prevalent and complex metabolic disorder closely associated with various complications, with significant implications for individual well-being and global healthcare systems. It is crucial to have a comprehensive understanding of the divergent trajectories of type 1 and type 2 diabetes and a firm grasp of these conditions' rising prevalence and underlying pathophysiological mechanisms. This knowledge is vital for effectively addressing the complex challenges presented by the widespread occurrence of diabetes [13]. The establishment of this knowledge serves as a foundation for exploring the complexities of cardiovascular complications associated with diabetes.

Microvascular complications associated with diabetes

Within the complex framework of complications associated with diabetes, it is crucial to acknowledge the significant role played by microvascular complications. These complications are primarily present in small blood vessels. They are characterized by their gradual onset and potential for significant organ damage [14]. This section will examine three significant microvascular complications: retinopathy, nephropathy, and neuropathy. Each of these aspects presents unique challenges, mechanisms, and management strategies, contributing to the intricate landscape of diabetes.

Retinopathy

Diabetic retinopathy is widely recognized as a significant and detrimental microvascular complication associated with diabetes. It impacts the fragile blood vessels within the retina, the light-sensitive tissue that lines the posterior part of the eye and is responsible for capturing visual images. Diabetic retinopathy is a progressive medical condition that commonly impacts both eyes and can cause substantial visual impairment or complete loss of vision if not adequately addressed [15]. The development of diabetic retinopathy is closely associated with long-term elevated blood glucose levels. Elevated blood glucose levels have been found to cause damage to the small blood vessels in the retina, which is called microangiopathy. Over time, this damage can lead to various clinical manifestations. Non-proliferative diabetic retinopathy (NPDR) is characterized by microaneurysms, retinal hemorrhages, and retinal exudates during its initial stages [16]. These alterations may manifest as asymptomatic or lead to mild visual impairments. Proliferative diabetic retinopathy (PDR) is a condition that may occur as the disease advances and is characterized by the proliferation of anomalous blood vessels on the surface of the retina. The vessels in question are delicate and susceptible to bleeding, resulting in vitreous hemorrhages. This stage is characterized by a higher degree of visual impairment [17]. Risk factors associated with the development of diabetic retinopathy encompass the duration of diabetes, suboptimal management of blood glucose levels, hypertension, dyslipidemia, and pregnancy. There is a possibility that genetic predisposition could contribute to an individual's vulnerability to retinopathy [17].

Treatment Options and Prevention Strategies

Laser photocoagulation: Laser therapy is a viable treatment option to occlude or eliminate abnormal blood vessels in the retina. This procedure effectively seals off any leakage from these vessels [16].

Anti-VEGF therapy: The administration of intravitreal injections containing vascular endothelial growth factor (VEGF) inhibitors has effectively mitigated the proliferation of abnormal blood vessels [16].

Vitrectomy is a surgical procedure that may be required in cases of advanced vitreous hemorrhage or retinal detachment. Prevention strategies emphasize the importance of maintaining glycemic control, managing blood pressure, and controlling lipid levels [13-15]. It is highly recommended to undergo regular eye examinations, even without symptoms, to detect retinopathy early, when treatment is most effective.

Nephropathy

Diabetic nephropathy is a microvascular complication that impacts the kidneys, leading to a gradual decline in their filtration capacity for waste products in the bloodstream. It is widely recognized as a primary contributor to the development of end-stage renal disease (ESRD) on a global scale, underscoring its significant clinical importance. The development of diabetic nephropathy involves multiple factors, with chronic hyperglycemia playing a significant role [18]. Over a prolonged period, elevated blood glucose levels can lead to the deterioration of the complex network of small blood vessels, known as glomeruli, in the kidneys. This can manifest as microalbuminuria, which refers to detecting small quantities of albumin in the urine and serves as an early indicator of kidney damage. Over time, this condition develops into overt proteinuria, characterized by elevated protein levels in the urine. This is considered a key diagnostic criterion for diabetic nephropathy [17]. The condition progresses through multiple stages.

Hyperfiltration: During this initial phase, there is an elevation in the glomerular filtration rate (GFR), which indicates the body's initial reaction to hyperglycemia [18].

Microalbuminuria: It is a significant diagnostic characteristic and an indicator of renal impairment. It has the potential to develop into overt proteinuria [19].

Overt proteinuria: During this stage, the renal function is compromised, resulting in the kidneys being unable to retain proteins effectively. Consequently, there is a notable increase in protein excretion in the urine [20].

Decreased glomerular filtration rate (GFR): The GFR initiates a decline, which signifies compromised renal function. Increased blood pressure commonly occurs during this stage [21].

End-stage renal disease (ESRD): This represents the ultimate stage of kidney dysfunction, characterized by a significant decline in kidney function that requires the implementation of renal replacement therapies, such as dialysis or kidney transplantation [22].

Exploration of Treatment Modalities and Potential Interventions

The management of diabetic nephropathy primarily revolves around slowing down the condition's progression and mitigating the risk of complications. Properly controlling hypertension is crucial to minimizing the risk of kidney damage. Healthcare professionals frequently prescribe medications such as ACE inhibitors and ARBs [22,23]. Lifestyle adjustments, such as adopting a low-sodium diet and engaging in regular exercise, can effectively contribute to managing blood pressure and enhancing overall health. Medications that inhibit the renin-angiotensin-aldosterone system (RAAS), such as ACE inhibitors and ARBs, are frequently employed to safeguard renal function. It is crucial to regularly monitor kidney function by conducting urine tests and estimating glomerular filtration rate (GFR) to identify early indications of nephropathy [24].

Multifaceted Challenge of Neuropathy in Diabetes

Diabetic neuropathy is a collective term used to describe a set of nerve disorders that occur due to nerve fiber damage caused by prolonged hyperglycemia. Diabetic neuropathy is a frequently encountered complication of diabetes that can impact multiple components of the nervous system, resulting in a diverse array of symptoms. Sensory neuropathy is a condition that primarily affects sensory nerves, resulting in symptoms such as numbness, tingling, burning sensations, or sharp pain. These symptoms typically begin in the feet and gradually spread to other body areas [25]. Autonomic neuropathy is a condition that impairs the normal functioning of the autonomic nervous system, which is responsible for regulating involuntary bodily functions. Common symptoms of this condition may include dizziness, an irregular heart rate, gastrointestinal disturbances, and bladder dysfunction. Peripheral neuropathy is a prevalent form that predominantly impacts the extremities, including the hands and feet. The condition may result in muscular weakness, diminished reflexes, and alterations in skin texture. The management of diabetic neuropathy is comprehensive, with the primary goals of symptom relief, disease progression deceleration, and enhancement of quality of life [26]. Achieving optimal blood glucose control is crucial for preventing and managing neuropathy. The implementation of strict glycemic control has the potential to decelerate or potentially cease the advancement of neuropathic symptoms. Medications such as tricyclic antidepressants, anticonvulsants, and specific antidepressants may be prescribed to manage neuropathic pain effectively [27].

Macrovascular complications of diabetes

The macrovascular complications associated with diabetes encompass a range of disorders that primarily impact the function of large blood vessels [25]. These complications present substantial risks to cardiovascular health and frequently result in severe consequences. This section will delve into three significant macrovascular complications: coronary artery disease (CAD), stroke, and peripheral artery disease (PAD) [26].

Coronary Artery Disease (CAD)

Coronary artery disease (CAD) is a prevalent macrovascular complication commonly associated with diabetes. This relationship encompasses various dimensions and incorporates several essential mechanisms. The presence of diabetes expedites the progression of atherosclerosis, a medical condition distinguished by the accumulation of lipid deposits (atherosclerotic plaques) within the coronary arteries [27]. These plaques have the potential to impede blood flow to the heart, resulting in angina or, in more severe instances, myocardial infarction (commonly known as a heart attack). Diabetic individuals are at a heightened risk of experiencing a myocardial infarction, commonly known as a heart attack. This increased risk can be attributed to various factors, such as atherosclerosis, endothelial dysfunction, and inflammation. In addition, individuals frequently encounter atypical symptoms, presenting a challenge in diagnosis [28]. Angina, a condition marked by chest pain or discomfort, is frequently observed in individuals with diabetes. This condition can be attributed to decreased blood flow to the myocardium due to coronary artery disease (CAD).

The risk factors for coronary artery disease (CAD) in individuals with diabetes include both traditional factors, such as hypertension, dyslipidemia, and smoking, and diabetes-specific factors, such as glycemic control and duration of diabetes. The importance of early detection cannot be overstated, and it is imperative to utilize stress tests, coronary angiography, and coronary calcium scoring as screening methods [29]. Management strategies include lifestyle modifications, such as adhering to a heart-healthy diet, exercising regularly, and discontinuing smoking. Medications, such as antiplatelet agents and statins, are essential in mitigating cardiovascular risk. In severe cases, it may be necessary to consider coronary revascularization procedures such as angioplasty and stent placement or coronary artery bypass surgery [30].

The Interplay Between Diabetes and Stroke: An Intricate Relationship

The presence of diabetes dramatically increases the likelihood of experiencing a stroke, thereby establishing it as a notable macrovascular complication. Multiple factors contribute to this increased risk. Ischemic strokes, which occur when a blood vessel supplying the brain becomes blocked, are more frequently observed in individuals with diabetes due to atherosclerosis and endothelial dysfunction. These strokes may lead to neurological impairments, which can vary depending on the specific brain region that is affected [12]. Hemorrhagic strokes involving bleeding into the brain are less prevalent but have been observed in individuals with diabetes. Inadequately managed blood pressure significantly contributes to this risk. The prevention of strokes in individuals with diabetes requires the implementation of comprehensive strategies. Blood glucose control is crucial for minimizing the risk of both ischemic and hemorrhagic strokes. The proper management of hypertension plays a critical role in preventing hemorrhagic strokes [13]. Healthcare professionals may prescribe medications such as ACE inhibitors and ARBs. Incorporating a heart-healthy lifestyle, encompassing a well-balanced diet, consistent physical activity, and smoking cessation, can effectively reduce the risk of stroke. Specific individuals with diabetes may experience potential advantages from using antiplatelet agents, such as aspirin, for primary or secondary stroke prevention. The effective management of stroke in individuals with diabetes requires specialized care, which encompasses various aspects such as rehabilitation, medication management, and the treatment of comorbid conditions like hypertension and hyperlipidemia [14].

Peripheral Arterial Disease (PAD)

Peripheral arterial disease (PAD) is a macrovascular complication associated with diabetes. Peripheral arterial disease (PAD) is characterized by the narrowing or blockage of arteries that supply blood to the limbs, typically caused by atherosclerotic plaques. Diabetes contributes to peripheral artery disease (PAD) through various mechanisms. Like coronary artery disease (CAD), diabetes expedites the progression of atherosclerosis in peripheral arteries, thereby diminishing blood circulation to the lower extremities [5-7]. Diabetic neuropathy has the potential to obscure the symptoms of PAD, as individuals with neuropathy may not manifest the usual pain or discomfort commonly associated with this condition. Peripheral arterial disease (PAD) commonly manifests with symptoms such as intermittent claudication (pain in the legs while walking), reduced blood flow to the extremities resulting in coldness, and the presence of non-healing ulcers or wounds on the feet [7]. The diagnostic procedures for this condition typically involve conducting ankle-brachial index (ABI) measurements, performing vascular imaging, and utilizing angiography. The potential complications of peripheral artery disease (PAD) in individuals with diabetes can be severe. These complications may include limb ischemia, gangrene, and the need for limb amputations. Timely identification and intervention are imperative to mitigate these consequences [8].

Potential Treatment Options

The management of peripheral artery disease (PAD) in individuals with diabetes entails the implementation of a multifaceted approach. Lifestyle changes such as quitting smoking, exercising regularly, and adopting a heart-healthy diet can effectively enhance blood circulation and alleviate symptoms. Prescribed medications, such as antiplatelet agents and statins, may be recommended to mitigate the potential for complications [9]. Endovascular procedures, such as angioplasty with or without stent placement, are effective interventions for addressing arterial blockages and restoring blood flow. Surgical interventions: In instances of significant severity, surgical bypass procedures or amputation may be deemed necessary. In summary, it is essential to acknowledge that macrovascular complications associated with diabetes present significant risks to cardiovascular health and overall well-being [10]. It is crucial to understand the complex interconnections between diabetes and conditions such as coronary artery disease (CAD), stroke, and peripheral artery disease (PAD) to effectively prevent, diagnose, and manage these health issues promptly. An essential strategy for addressing macrovascular challenges in individuals with diabetes involves a multidisciplinary approach encompassing glycemic control, lifestyle modifications, and medication management [11].

Pathophysiological mechanisms that establish a connection between diabetes and cardiovascular complications

Diabetes mellitus is a multifaceted metabolic disorder with persistently high blood sugar levels. This condition substantially increases the likelihood of developing cardiovascular complications, which can damage small and larger blood vessels. These complications are not isolated incidents but the outcome of complex pathophysiological mechanisms [12]. This extensive analysis will thoroughly investigate the intricate mechanisms linking diabetes to cardiovascular complications and explore potential therapeutic approaches. Hyperglycemia as a trigger for oxidative stress hyperglycemia, a condition characterized by high blood sugar levels, has been identified as a significant factor in the development of oxidative stress [13]. Hyperglycemia, a prominent characteristic of diabetes, is a pivotal factor in the initiation and progression of oxidative stress. When glucose levels persistently remain elevated, glucose molecules engage in non-enzymatic glycation reactions with proteins and lipids, forming advanced glycation end-products (AGEs). Advanced glycation end products (AGEs) initiate physiological reactions, producing oxidative stress [14].

Oxidative Stress: Interconnection Between Microvascular and Macrovascular Damage

Oxidative stress occurs when there is a disparity between the generation of reactive oxygen species (ROS) and the protective mechanisms of antioxidants within the body. In the context of diabetes, it is widely recognized that oxidative stress plays a significant role in developing microvascular and macrovascular complications. Microvascular damage occurs within the microvasculature, where oxidative stress adversely affects small blood vessels, leading to compromised blood flow and hindered nutrient delivery to diverse tissues [15]. This condition contributes to diabetic retinopathy, nephropathy, and neuropathy. Numerous studies have indicated that antioxidants, such as vitamins C and E, can alleviate microvascular complications by diminishing oxidative stress. Oxidative stress has been observed to have detrimental effects on the integrity of significant blood vessels, thereby facilitating the development of atherosclerotic plaques [16]. These plaques cause a narrowing of the arteries, which leads to a restriction in blood flow and an elevated risk of myocardial infarction and stroke. The potential use of antioxidant therapies to address oxidative stress in macrovascular complications has been investigated, yielding varying outcomes [17].

Chronic Inflammation: A Persistent Co-Occurrence With Diabetes

Chronic inflammation is a prominent characteristic of diabetes and plays a substantial role in developing vascular complications. Persistent hyperglycemia elicits immune responses that result in the synthesis of pro-inflammatory cytokines and chemokines. Prolonged inflammation can potentially impair the integrity of the intricate blood vessels found in the retina, kidneys, and nerves, thereby playing a role in developing diabetic retinopathy, nephropathy, and neuropathy. Ongoing research explores therapeutic approaches that address inflammation, including non-steroidal anti-inflammatory drugs (NSAIDs) and innovative biologics [18]. These strategies are being investigated for their potential to mitigate microvascular damage. The role of inflammation in atherosclerosis, a significant contributor to coronary artery disease and stroke, is vital for macrovascular complications. Inflammatory cells have been observed to infiltrate atherosclerotic plaques, compromising their stability and elevating the likelihood of rupture [18]. Anti-inflammatory interventions, such as statins and emerging biologic agents like canakinumab, are implemented to mitigate inflammation and lower the risk of cardiovascular complications. A precursor to vascular disruption endothelial dysfunction is a condition that serves as an early indicator of potential disturbances within the vascular system. Endothelial dysfunction is a significant factor in developing vascular complications associated with diabetes. It is characterized by a decrease in the availability of nitric oxide (NO) and an increase in the production endothelin-1 [19].

Microvascular complications arise due to impaired nitric oxide (NO) production, which disrupts the intricate equilibrium between vasoconstriction and vasodilation in small blood vessels. The management of microvascular damage has shown promise through the utilization of medications such as angiotensin-converting enzyme (ACE) inhibitors and angiotensin receptor blockers (ARBs), which specifically target endothelial health [20]. Macrovascular complications arise due to endothelial dysfunction, which plays a significant role in the development of atherosclerosis by facilitating vasoconstriction, inflammation, and oxidative stress. Therapeutic interventions that specifically address endothelial function, such as nitric oxide (NO) donors and endothelin receptor antagonists, are designed to promote the restoration of vascular health and mitigate the risk of macrovascular complications. Researchers are investigating novel therapeutic approaches targeting endothelial function [21]. These therapies include sodium-glucose cotransporter 2 (SGLT2) inhibitors, which have demonstrated cardiovascular benefits by enhancing endothelial health and mitigating oxidative stress.

In summary, the pathophysiological mechanisms that connect diabetes to cardiovascular complications are complex and involve various factors such as oxidative stress, chronic inflammation, and endothelial dysfunction. These interrelated processes play a role in causing damage to both microvascular and macrovascular systems, affecting multiple organs and systems within the body [23]. Although there is promise in managing complications associated with diabetes through the use of antioxidants, anti-inflammatory agents, and therapies that target endothelial health, it is imperative to conduct further research to enhance treatment strategies and ultimately improve outcomes for individuals living with this condition. Recognizing these mechanisms highlights the importance of implementing comprehensive, multifaceted strategies for managing diabetes and preventing its severe cardiovascular complications [22-26].

Risk assessment for cardiovascular complications in diabetics

Diabetes mellitus is widely recognized as a significant risk factor for developing various cardiovascular complications, such as coronary artery disease, stroke, and peripheral arterial disease [23]. Considering the profound implications of these complications, it is imperative to prioritize risk assessment as a fundamental aspect of diabetes management. This section explores the significance of risk assessment tools, the role of biomarkers and imaging techniques, and the potential benefits of early monitoring in enhancing outcomes and informing treatment decisions [25].

Significance of Risk Assessment Tools

Risk assessment tools are crucial in effectively identifying individuals with diabetes at an increased risk of developing cardiovascular complications. These tools enable healthcare providers to customize interventions and preventive strategies based on each patient's needs [26].

The Framingham risk score is a commonly utilized tool for assessing an individual's 10-year risk of developing coronary heart disease. The model includes risk factors such as age, gender, smoking status, blood pressure, cholesterol levels, and diabetes [25].

The ASCVD risk calculator was developed by the American College of Cardiology and the American Heart Association. Its purpose is to assess an individual's risk of developing atherosclerotic cardiovascular disease (ASCVD). The factors considered include age, gender, race, cholesterol levels, blood pressure, diabetes status, and smoking history [27].

The UKPDS Risk Engine is a tool utilized for assessing the risk of developing cardiovascular disease in individuals. The UK Prospective Diabetes Study (UKPDS) Risk Engine is a specialized tool developed to assess the risk of coronary heart disease in individuals diagnosed with diabetes [12]. The model considers diabetes-specific variables such as HbA1c, duration of diabetes, and blood pressure.

Utilization of Biomarkers, Imaging Techniques, and Diagnostic Tools

Biomarkers, imaging techniques, and diagnostic tools provide significant insights into cardiovascular risk. They can assist in the assessment of risk for individuals with diabetes. Biomarkers are measurable indicators that can be used to assess various biological processes or conditions [14]. C-reactive protein (hs-CRP) is a biomarker linked to an increased risk of cardiovascular disease when found at elevated levels. It is an indicator of inflammation and can assist in evaluating risk and making treatment decisions. B-type natriuretic peptide (BNP) and N-terminal pro-B-type natriuretic peptide (NT-proBNP) indicate cardiac stress. Elevated levels of specific markers may indicate the presence of heart failure or an increased risk of cardiovascular complications [12]. Coronary CT angiography is a non-invasive imaging technique that evaluates coronary artery disease by visually representing coronary artery anatomy and identifying the presence of plaques and stenoses. Constraint induced movement therapy (CIMT) measurements can evaluate subclinical atherosclerosis in the carotid arteries, offering valuable insights into an individual's cardiovascular risk. Myocardial perfusion imaging (MPI) is a diagnostic technique utilized to evaluate myocardial blood flow and detect ischemia, aiding in identifying coronary artery disease [16].

Additional Diagnostic Tools

Electrocardiography (ECG) is a diagnostic tool to identify cardiac abnormalities, including arrhythmias, ischemia, and other conditions. It plays a crucial role in assessing the risk of cardiovascular complications. Echocardiography is a medical imaging technique used to evaluate the structure and function of the heart. It is employed to identify various cardiac conditions, including left ventricular hypertrophy and diastolic dysfunction. The timely surveillance of cardiovascular risk factors in individuals with diabetes is crucial for enhancing outcomes and informing treatment strategies. The proactive approach enables timely intervention and the implementation of preventive measures [17]. Blood glucose control is an essential aspect of managing diabetes. It involves monitoring and regulating blood sugar levels to maintain optimal health. Hemoglobin A1c Monitoring: Regular monitoring of hemoglobin A1c (HbA1c) levels offers valuable information regarding managing blood glucose over an extended period. There is a correlation between maintaining tight glycemic control and a decrease in cardiovascular risk. Continuous glucose monitoring (CGM) systems provide individuals with diabetes with real-time data on their glucose levels, enabling them to modify their treatment regimens as necessary promptly [18].

Effective Blood Pressure Management

Promoting home blood pressure monitoring among individuals with diabetes is highly encouraged, as it facilitates improved control and early hypertension detection [18]. Ambulatory blood pressure monitoring (ABPM) is a diagnostic method for comprehensively assessing blood pressure patterns over 24 hours. It plays a crucial role in the diagnosis and management of hypertension.

Lipid Profile Evaluation

Routine evaluations of lipid profiles, encompassing measurements of total cholesterol, low-density lipoprotein (LDL) cholesterol, high-density lipoprotein (HDL) cholesterol, and triglycerides, inform the decision-making process for lipid-lowering therapy. The measurement of apolipoproteins and lipoprotein subclasses provides a comprehensive evaluation of cardiovascular risk, offering more detail. It is imperative to regularly monitor serum creatinine and estimated glomerular filtration rate (eGFR) to assess renal function, as compromised kidney function poses a risk for cardiovascular complications [20]. The urine albumin-to-creatinine ratio (UACR) is a diagnostic measure for kidney health. An elevated UACR level is indicative of early kidney damage and may serve as an indicator of heightened cardiovascular risk. In summary, risk assessment tools, biomarkers, imaging techniques, and early monitoring are crucial in managing cardiovascular risk in individuals with diabetes. These tools facilitate healthcare providers in identifying individuals who are at a heightened risk of experiencing cardiovascular complications [24]. Additionally, they allow for the customization of interventions based on individual needs and the monitoring of treatment strategies to assess their effectiveness. Timely surveillance, in conjunction with suitable interventions, has the potential to considerably enhance results and inform treatment choices, ultimately lessening the impact of cardiovascular disease in individuals with diabetes [25].

Cardiovascular complications pose a significant concern among individuals diagnosed with diabetes. Preventive and management strategies involve lifestyle modifications, pharmacological interventions, medications, and therapies. This comprehensive section delves into the influence of diet, exercise, and weight management on cardiovascular complications related to diabetes. It presents evidence-based lifestyle recommendations and provides insights into pharmacological interventions for managing diabetes [16]. These interventions include medications for controlling blood sugar levels, using cardiovascular risk, and using antiplatelet agents, statins, and other pharmaceuticals [24].

Prevention and management strategies for diabetes-related cardiovascular complications

Lifestyle Modifications: Dietary Adjustments, Physical Activity, and Weight Control

A heart-healthy diet is essential for preventing cardiovascular complications in individuals with diabetes. It impacts blood glucose regulation, lipid profiles, and blood pressure. Maintaining a balanced diet that includes a variety of whole grains, fruits, vegetables, lean proteins, and healthy fats is recommended. Reducing the consumption of processed foods, sugary beverages, and excessive amounts of salt is imperative [23]. Emphasizing portion control and carbohydrate management is crucial for individuals to manage their blood glucose levels effectively. Regular physical activity is essential for maintaining cardiovascular health and managing glycemic control. Promoting aerobic exercises, such as brisk walking, cycling, and swimming, is recommended for at least 150 minutes weekly. Engaging in strength training exercises can effectively contribute to the development of muscle mass and enhance metabolic function. Moreover, engaging in yoga or Tai Chi activities can enhance flexibility and balance, mitigating the likelihood of experiencing falls [26].

Weight management: Obesity is a notable risk factor for cardiovascular complications in individuals with diabetes. Weight management strategies encompass attaining a caloric equilibrium through a dual approach involving caloric reduction through dietary adjustments and augmented caloric expenditure through engagement in physical activity [29]. Behavioral support effectively addresses the psychological factors that influence weight. In cases of severe obesity and diabetes, bariatric surgery may be warranted to achieve significant weight loss.

Evidence-Based Lifestyle Recommendations

Carbohydrate management: Promote the practice of carbohydrate counting as a means to assist individuals with diabetes in effectively managing their blood glucose levels. It is recommended to consume complex carbohydrates that have a low glycemic index [2].

Promotion of unsaturated fats: Encourage the incorporation of unsaturated fats, such as those present in olive oil, nuts, and fatty fish, into one's diet while concurrently limiting the intake of saturated and trans fats [3].

Behavioral counseling: We provide behavioral counseling services to address emotional eating, binge eating, and other psychological factors that may impact an individual's weight [3].

Pharmaceutical Interventions and Therapeutic Approaches for Diabetes Management

Metformin is commonly prescribed as the initial medication for the treatment of diabetes. It effectively enhances insulin sensitivity and decreases glucose production in the liver. Sulfonylureas are a class of medications that effectively stimulate insulin release from pancreatic beta cells. Dipeptidyl peptidase-4 (DPP-4) inhibitors are pharmaceutical agents that can enhance insulin secretion and reduce glucagon release [24]. GLP-1 receptor agonists: Glucagon-like peptide-1 receptor agonists can enhance insulin secretion, inhibit glucagon production, and reduce weight. SGLT2 inhibitors, also known as sodium-glucose cotransporter-2 inhibitors, are pharmaceutical agents that lower blood glucose levels by facilitating glucose excretion through the kidneys. Additionally, these inhibitors have been shown to impact reducing cardiovascular events positively [5-10].

Strategies for Reducing Cardiovascular Risk: Antiplatelet Agents, Statins, and Beyond

Antiplatelet agents: It is recommended to administer low-dose aspirin for primary prevention of cardiovascular events in individuals with diabetes who are at high risk. In the context of secondary prevention, it may be advisable to consider dual antiplatelet therapy following a myocardial infarction or stroke [23]. Statins are widely recognized as essential medications to reduce LDL cholesterol levels and mitigate cardiovascular risk. It is generally recommended to prescribe high-intensity statin therapy for individuals with diabetes and a confirmed diagnosis of cardiovascular disease [25]. 

Additional lipid-lowering agents: In cases where statin therapy is either not well-tolerated or does not yield satisfactory results, alternative lipid-lowering agents such as ezetimibe or PCSK9 inhibitors may be considered [20].

Blood pressure control: A proactive approach to managing blood pressure is recommended, aiming for target values below 130/80 mm Hg to mitigate cardiovascular risk. Certain medications, such as GLP-1 receptor agonists and SGLT2 inhibitors, have exhibited cardiovascular benefits beyond glycemic control. These agents are recommended for individuals diagnosed with diabetes with a pre-existing cardiovascular disease [21]. Ongoing research is investigating novel therapies to reduce cardiovascular risk in individuals with diabetes. These therapies include cardiovascular outcome trials (CVOTs) and gene-based therapies. In summary, preventing and managing cardiovascular complications in individuals with diabetes requires a holistic approach. Implementing lifestyle modifications that prioritize dietary adjustments, regular physical activity, and effective weight management strategies is crucial for enhancing cardiovascular outcomes [22]. Pharmacological interventions aimed at glycemic control and reducing cardiovascular risks, such as antiplatelet agents, statins, and other lipid-lowering drugs, are paramount in managing cardiovascular risk. The implementation of personalized care plans that incorporate these strategies has the potential to significantly alleviate the impact of cardiovascular disease on individuals with diabetes, ultimately enhancing their overall quality of life [23].

The global impact of diabetes mellitus as a significant health issue persists, and there is a growing body of knowledge regarding its cardiovascular complications [24]. The latest research findings provide valuable insights into the complex mechanisms that contribute to these complications and present promising prospects for developing innovative strategies for prevention and treatment. This section concisely overviews recent research findings, ongoing studies, and potential breakthroughs. It also highlights the importance of personalized medicine and precision interventions [25].

Current research and future directions in diabetes-related cardiovascular complications

Recent studies have contributed to a more comprehensive comprehension of the pathophysiological mechanisms that establish a connection between diabetes and cardiovascular complications. The topic of discussion pertains to inflammation and immune responses. Recent research has elucidated the significant role that chronic inflammation and immune responses play in developing diabetic atherosclerosis. Research findings suggest that the strategic targeting of specific inflammatory pathways has the potential to mitigate cardiovascular risk [13]. The gut microbiome's influence on cardiovascular health in individuals with diabetes is being investigated through emerging research on the microbiome and gut-heart axis. The presence of gut dysbiosis and microbial metabolites has been observed to contribute to inflammation and atherosclerosis development. Epigenetics is a field of study that investigates the various modifications that occur to DNA, such as DNA methylation and histone acetylation. These modifications have been found to impact the development of cardiovascular complications associated with diabetes significantly [17-20]. The comprehension of epigenetic alterations has the potential to pave the way for innovative therapeutic strategies.

Advancements in Cardiovascular Imaging

Recent advancements in the field of cardiovascular imaging have significantly improved our capacity to identify and closely monitor complications associated with cardiovascular health [24-28]. High-resolution coronary CT angiography facilitates the non-invasive evaluation of coronary artery disease, thereby assisting in the timely identification and classification of risk. Magnetic resonance imaging (MRI) is a medical imaging technique that offers comprehensive insights into cardiac structure and function. It plays a crucial role in detecting and analyzing subtle changes indicative of heart disease associated with diabetes [30,31].

Individualized Risk Assessment

Recent studies have underscored the significance of individualized risk assessment.

Genetic profiling: Through genetic studies, specific genetic variants have been identified that are associated with an elevated risk of cardiovascular complications in individuals with diabetes [7-9]. Personalized genetic risk assessments have the potential to inform and guide preventive strategies. Utilizing machine learning algorithms and predictive models involves the analysis of individual patient data to estimate the risk of cardiovascular events. These models can improve risk stratification and inform treatment decisions [10-12].

Current Research and Prospective Advancements

Cardiovascular outcome trials (CVOTs) are clinical studies conducted to assess the impact of specific interventions or treatments on cardiovascular outcomes. Ongoing cardiovascular outcome trials (CVOTs) are currently being conducted to examine the cardiovascular impact of anti-hyperglycemic agents further. The Semaglutide Cardiovascular Outcomes Trial (SELECT) evaluates the cardiovascular safety and efficacy of semaglutide, a GLP-1 receptor agonist [14]. This trial focuses on individuals with type 2 diabetes who already have established cardiovascular disease. The Dapagliflozin Effect on Cardiovascular Events (DECLARE-TIMI 58) trial is designed to assess the impact of dapagliflozin, an SGLT2 inhibitor, on cardiovascular outcomes in individuals with diabetes who have existing cardiovascular disease or are at risk for it [16].

Advanced Therapies and Targets

Several potential therapies and targets are currently being investigated for their promising outcomes. Ongoing research is currently investigating the potential of stem cell-based therapies in cardiac cell therapy. The aim is to repair damaged cardiac tissue and enhance heart function in individuals with diabetes [18]. The modulation of inflammatory pathways is currently being investigated as a potential strategy to mitigate cardiovascular risk in individuals with diabetes. One area of focus is the development of agents that specifically target interleukin-1β to inhibit its activity. These agents are being studied to determine their effectiveness in reducing cardiovascular risk in individuals with diabetes [20].

Precision medicine in lipid management: Precision-based approaches customize lipid-lowering therapy by considering individual patient profiles, encompassing genetic factors and lipid subtypes. Integrating genetic risk profiles into cardiovascular risk assessments enables the implementation of focused interventions for individuals with elevated genetic risk. Machine learning algorithms can analyze extensive datasets to identify personalized risk factors, thereby facilitating the implementation of customized preventive strategies [22]. Pharmacogenomics is a field of study that focuses on comprehending an individual's genetic response to medications to inform the selection of therapies that are both effective and safe. Precision medicine enables the customization of treatment plans according to an individual patient's distinct genetic, metabolic, and clinical attributes. The concept of patient-centered care is a fundamental principle in healthcare. It emphasizes placing the patient at the center of all [25].

Shared decision-making: Personalized medicine facilitates shared decision-making, empowering patients to actively make informed choices regarding their care and treatment. Adverse events can be mitigated by identifying individuals at a higher risk for medication-related complications [23]. By implementing personalized approaches, treatment-related adverse events can be minimized. In summary, recent research on diabetes-related cardiovascular complications has contributed to our understanding of pathophysiological mechanisms, advanced imaging techniques, and personalized risk assessment. Current research endeavors offer the potential for significant advancements in prevention and treatment, with a particular emphasis on precision interventions and personalized medicine [15]. By embracing these advancements, healthcare providers can provide individualized, patient-centered care that effectively mitigates the burden of cardiovascular complications in diabetes and enhances overall cardiovascular health outcomes [13-15].

This narrative review has explored the complex relationship between cardiovascular complications and diabetes, highlighting the significant contributions of both microvascular and macrovascular pathways [12-14]. As we bring this journey to a close, we will summarize the essential findings and insights obtained from investigating these pathways, highlighting their significant implications for diabetes. Furthermore, we emphasize the significance of ongoing research and the utilization of multidisciplinary approaches to tackle this intricate matter effectively [14-20].

Key findings and insights

Microvascular complications are a significant consequence of diabetes, resulting in a combination of three complications: retinopathy, nephropathy, and neuropathy. Diabetic retinopathy is the primary cause of blindness among adults in the working-age population, emphasizing the imperative of timely identification and intervention [12]. Diabetic nephropathy, characterized by the gradual deterioration of kidney function, is a substantial factor in the development of end-stage renal disease. Diabetic neuropathy comprises a range of types, each presenting with distinct symptoms, thereby requiring individualized approaches to management [15].

Macrovascular complications: Diabetes has a significant influence on the macrovascular system, leading to an increased susceptibility to coronary artery disease, stroke, and peripheral arterial disease. Atherosclerosis, a prevalent complication, arises from intricate interactions encompassing hyperglycemia, oxidative stress, inflammation, and endothelial dysfunction. Prioritizing the early detection and proactive management of macrovascular risk factors is crucial [13]. The pathophysiological mechanisms underlying diabetes-related cardiovascular complications are complex and multifaceted. Hyperglycemia plays a significant role in promoting oxidative stress, which in turn contributes to the development of microvascular and macrovascular damage. Chronic inflammation contributes to worsening vascular dysfunction, increasing the likelihood of complications [15]. Endothelial dysfunction, characterized by compromised nitric oxide bioavailability, is a significant factor in both microvascular and macrovascular scenarios [17].

Risk assessment and monitoring: It is crucial to utilize practical risk assessment tools to identify individuals with diabetes at an increased risk of developing cardiovascular complications. Biomarkers, advanced imaging techniques, and predictive models are instrumental in risk stratification [18]. Timely intervention and prevention necessitate the early monitoring of blood glucose, blood pressure, and lipid profiles.

Prevention and management strategies: Lifestyle modifications, including dietary adjustments, regular exercise, and effective weight management, are crucial in reducing cardiovascular risk. Pharmacological interventions are paramount in achieving glycemic control, managing lipid levels, and controlling blood pressure [23]. Antiplatelet agents and statins are essential medications for mitigating cardiovascular risk. Novel therapeutic approaches targeting inflammation and promoting endothelial health show tremendous potential [24].

Personalized medicine: Recognizing the heterogeneity among individuals with diabetes has led to the increasing prominence of personalized medicine and precision interventions. Genetic profiling, machine learning, and therapeutic tailoring facilitate the development of personalized risk assessment and treatment plans for individuals [25]. The implementation of a patient-centered approach aims to maximize outcomes while minimizing the occurrence of adverse events.

Importance of Microvascular and Macrovascular Pathways

Gaining a comprehensive understanding of the complexities associated with microvascular and macrovascular pathways in diabetes is essential for various reasons [25].

Comprehensive care: The effective management of diabetes requires a holistic approach that encompasses a thorough understanding of both microvascular and macrovascular complications. Failing to prioritize both aspects may result in inadequate care and less-than-optimal results [23].

Early intervention: Microvascular and macrovascular complications frequently present subtly, underscoring the significance of timely identification and intervention. Identifying the connections between hyperglycemia, oxidative stress, inflammation, and endothelial dysfunction enables the prompt implementation of preventive strategies [5].

Customization of treatment: Personalized medicine necessitates a comprehensive understanding of the intricate mechanisms involved in cardiovascular complications associated with diabetes. Customizing treatment plans based on individual patient profiles maximizes effectiveness and reduces adverse effects [29]. Effective management of cardiovascular complications in diabetes necessitates the establishment of multidisciplinary collaboration. Endocrinologists, cardiologists, nephrologists, ophthalmologists, and other specialized medical professionals must collaborate effectively to deliver comprehensive healthcare services. Promoting and providing assistance for ongoing research initiatives that investigate pathophysiological mechanisms, identify novel therapeutic targets, and explore innovative treatment modalities. Cardiovascular outcome trials, genetic studies, and biomarker research demonstrate significant potential [30].

Promote the implementation of multidisciplinary care approaches that facilitate the integration of specialists from diverse disciplines, fostering a comprehensive and patient-centric approach to healthcare delivery. Multidisciplinary teams can effectively address the diverse needs of individuals experiencing complications related to diabetes and cardiovascular conditions [25].

Patient education and empowerment: Our goal is to empower individuals with diabetes to take an active role in their healthcare. We achieve this by providing comprehensive education on risk factors, lifestyle modifications, and the importance of adhering to prescribed medications. Well-informed patients are more inclined to actively participate in preventive measures [17].

Policy and guidelines: It is recommended to advocate for implementing policies and guidelines that emphasize cardiovascular risk assessment and management within the context of diabetes care [19]. It is imperative to prioritize cardiovascular health within healthcare systems.

Global initiatives: Foster international collaboration to address the widespread prevalence of diabetes and its associated cardiovascular complications. The exchange of best practices and research findings can expedite progress [20].

## Conclusions

In conclusion, the complex network of cardiovascular complications associated with diabetes, encompassing both microvascular intricacies and significant macrovascular consequences, highlights the pressing necessity for a comprehensive approach to diabetes management. In this narrative review, we have explored the intricate interplay of metabolic dysregulation, inflammation, oxidative stress, and endothelial dysfunction, all of which contribute significantly to the development of these complications. The primary recommendation derived from this review is evident: diabetes care should be individualized, proactive, and consistent, with healthcare professionals adapting their methods to suit each patient and emphasizing early intervention and preventive measures. As we navigate the continuously evolving realm of diabetes and its associated cardiovascular complications, it is crucial to acknowledge the significant influence these conditions exert on the lives of countless individuals. By acknowledging the interdependence of micro and macrovascular pathways, embracing recent advancements in research and technology, and adopting a patient-centric approach, we can strive towards a future where the impact of cardiovascular complications in diabetes is significantly reduced, ultimately resulting in enhanced quality of life for individuals affected by this prevalent and demanding condition.
